# Novel Hybrid Peptide Cecropin A (1–8)-LL37 (17–30) with Potential Antibacterial Activity

**DOI:** 10.3390/ijms17070983

**Published:** 2016-06-29

**Authors:** Xu-Biao Wei, Ru-Juan Wu, Da-Yong Si, Xiu-Dong Liao, Lu-Lu Zhang, Ri-Jun Zhang

**Affiliations:** Laboratory of Feed Biotechnology, State Key Laboratory of Animal Nutrition, College of Animal Science and Technology, China Agricultural University, Beijing 100193, China; Weixubiao01@126.com (X.-B.W.); lingxiabenpao@aliyun.com (R.-J.W.); lionsdy1@gmail.com (D.-Y.S.); liaoxd56@163.com (X.-D.L.); zhanglulu09@126.com (L.-L.Z.)

**Keywords:** hybrid peptide, cecropin A (1–8)-LL37 (17–30), antibacterial activity, hemolytic activity, synergistic interaction

## Abstract

Hybridizing different antimicrobial peptides (AMPs) is a particularly successful approach to obtain novel AMPs with increased antimicrobial activity but minimized cytotoxicity. The hybrid peptide cecropin A (1–8)-LL37 (17–30) (C-L) combining the hydrophobic N-terminal fragment of cecropin A (C) with the core antimicrobial fragment of LL37 (L) was designed and synthesized. C-L showed higher antibacterial activity against all indicator strains than C and L, and no hemolytic activity to sheep erythrocytes was observed. C-L kills bacterial cells and causes disruption of surface structure, as determined by scanning electron microscopy. Synergistic effects were observed in the combination of C-L with several antibiotics (chloramphenicol, thiamphenicol, or neomycin sulfate) against *Escherichia coli* and *Staphylococcus aureus*.

## 1. Introduction

The increasing microbial resistance to conventional antibiotics is a universal public concern [1], which results in great efforts to develop new safe therapeutic agents [2,3]. Antimicrobial peptides (AMPs) are attracting more and more attention as a therapeutic alternative in the field of disease control and prevention. AMPs are naturally distributed short amphipathic cationic molecules with a unique mechanism of action and broad spectrum of antimicrobial activity [4,5,6]. Many AMPs, such as melittin, human cathelicidin cationic antimicrobial peptide LL37, indolicidin, and IsCT, show undesirable hemolytic activity toward eukaryotic cells while killing microbes, which largely compromised their development as pharmaceuticals [2,7,8]. Hybridizing different AMPs has been demonstrated to be an effective method to avoid these innate limitations [9,10]. Additionally, the combination of antibiotics with proteinaceous AMPs is a good option to enhance the antibacterial activity and eliminate the adverse effects [11].

Cecropin A (C), produced by *Hyalophora cecropia*, is a linear helical antibacterial peptide composed of 37 amino acid residues. It is divided into an N-terminal amphipathic α-helix domain and C-terminal hydrophobic α-helix domain. C shows broad bioactivities, such as antibacterial, anti-inflammation, and anticancer activities, but does not lyse eukaryotic cells [12,13,14,15]. The N-terminal amphipathic α-helix domain corresponding to the first 7–8 residues has been widely applied to design a large group of novel peptides, such as CA (1–8)-M (1–12) [10,16], CA (1–8)-M (1–18) [17], CA (1–8)-MA (4–12) [9], CA (1–7)-M (1–13) [10], and CA (1–7)-M (2–9) [18], by hybridizing with segments from melittin whose hemolytic concentration was extremely lower than its minimal inhibitory concentrations (MICs). Antimicrobial studies on these hybrid peptides showed that their lethal concentrations against a panel of bacteria ranged from 0.1 to 15 µM, while hemolytic concentrations were >300 µM [10,17,19,20].

The human cathelicidin cationic antimicrobial peptide LL-37 (L), which is a linear helical peptide containing 37 amino acid residues, is isolated from human leukocytes and epithelia. L plays a critical role in the process of antimicrobial infection, angiogenesis, wound healing, and apoptosis [7,21,22,23,24]. However, it shows undesirable hemolytic activity against eukaryotic cells by inducing membrane disruption at concentrations of 25–30 µM, which is slightly higher than its MIC value. This property greatly limits its clinical application [24]. Numerous antimicrobial studies on a series of L fragments showed that a well-defined helical structure in the region corresponding to residues 17–30 was responsible for its antimicrobial, anticancer, and antiviral activities [7,25,26].

Recently, many studies suggested that net positive charge and helical content are important factors to the antibacterial activity of AMPs [27,28]. Early studies from our laboratory also demonstrated the importance of helical structure to the antibacterial activity of AMPs [9]. In the present study, the hybrid peptide C (1–8)-L (17–30) (C-L) was designed by combining the N-terminal amphipathic α-helix fragment of C with the core antimicrobial fragment of L. This approach was on the basis of the hypothesis that the combination will elevate the antimicrobial property and minimize the hemolytic activity by forming more net positive charges and higher content of helical structure. Positive charge was believed to be closely related to the electrostatic bonding force between the AMPs and the negatively charged bacterial cell membrane [29,30]. Helical structure could promote deep insertion of AMPs into bacterial cell membrane [31,32]. In order to pave the way for its clinical application, various tests, including antibacterial activity, hemolytic activity, time-killing curve (TKC), and bacterial morphological changes, were carried out in vitro to evaluate the antibacterial efficacy of C-L.

## 2. Results

### 2.1. Primary Structural Parameters of Peptides

**Figure 1:**
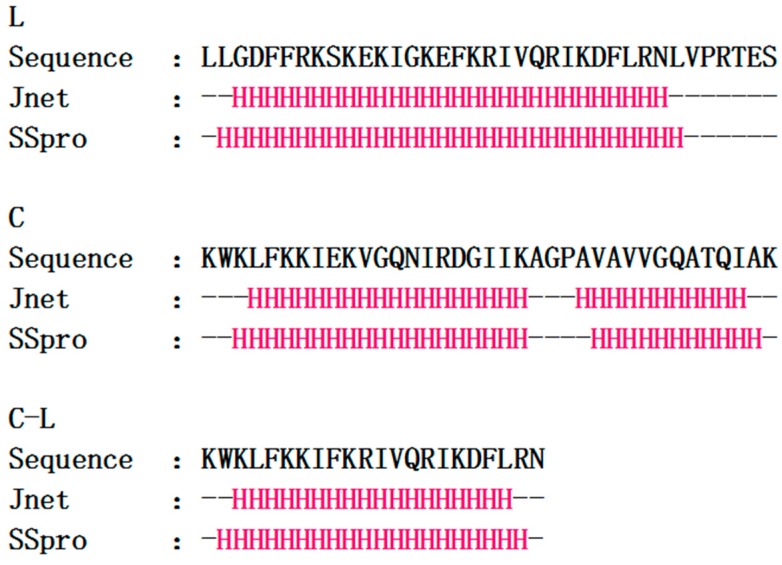
The secondary structure of hybrid peptides predicted by Jpred 4 and SSpro. “H” in red represents α-helical structure and “–” represents non α-helical structure.

As shown in Table 1, Matrix-assisted laser desorption ionization (MALDI) mass spectrometry indicated the purity of final peptides products were all above 95%. The amino acid sequence and molecular weight were confirmed to be correct. The molecular modeling results showed the secondary structure of C-L differed from that of the parental peptides. In comparison with C and L, C-L had a higher net charge (+8) and contained a higher concentration of helical residues (90.9%) (Table 2). C and C-L had the same hydrophobic residue concentration (45%), and this was higher than that of L (35%). The predicted secondary structural type of each residue (Figure 1) showed that C-L had a higher propensity for helical structure than L and C, particularly in the analysis of SSpro [33], 20 amino acids of C-L took the form of a helical structure, accounting for 90.9% of the total amino acid residues, which was greater than both L (81.1%) and C (81.1%).

### 2.2. Antibacterial and Hemolytic Activity

The antibacterial and hemolytic activity (HC_50_) of the peptides were determined through micro-broth dilution method (Table 3). When compared to parental peptides, the antibacterial activity of C-L was greatly improved. Results showed that all the six indicator strains were more susceptible to C-L (MICs ranged 2–16 µg/mL) than C (MICs ranged 4.0–198 µg/mL) and L (MICs ranged 13–90 µg/mL) (*p* < 0.01). C-L treated group showed lower HC_50_ to sheep erythrocyte cells than the C-treated group and L-treated group (*p* < 0.01). C-L did not show cytotoxicity (HC_50_ was 221 µg/mL, HC_50_ > 128 µg/mL was determined as no cytotoxicity) while the HC_50_ of L and C was 32 and 169 µg/mL, respectively.

### 2.3. Sterilization Speed and Efficacy

Both *Escherichia coli* (*E.* *coli*) CVCC 245 and S*taphylococcus aureus* (*S. aureus*) ATCC 25923 were susceptible to the peptides tested. Table 4 showed the sterilization rate of L, C, and C-L on *S. aureus* ATCC 25923 and *E.* *coli* CVCC 245. For *S. aureus* ATCC 25923, C-L caused 57% viability decrease at 1 h, while L and C caused about 10% and 46% decreases, respectively. The sterilization rate of C-L remained the highest in the following 4 h. For *E. coli* CVCC 245 the survival rate to C-L at 1, 2, and 3 h were 29%, 16%, and 5%, respectively. However, the strain survival rate was 44%, 35%, and 14% at 1, 2, and 3 h, respectively, when L was used as a bactericide, and 44%, 30%, and 15%, respectively, when C was used (Table 4). No matter *S. aureus* or *E.* *coli*, the C-L-treated group showed lower viability than the C-treated group and L-treated group at 1, 2, and 3 h (*p* < 0.05). According to the results shown in Table 4, C-L was found to be more effective than C or L in sterilization, especially in the first 3 h.

### 2.4. Disruption of Bacterial Cell Surface Structure Integrity

**Figure 2:**
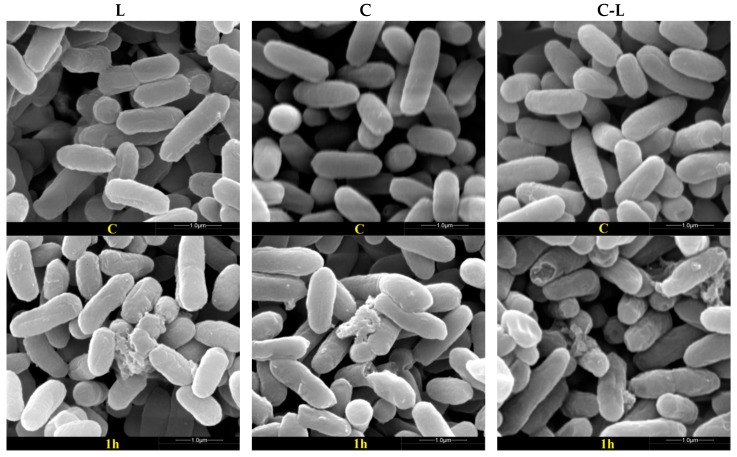

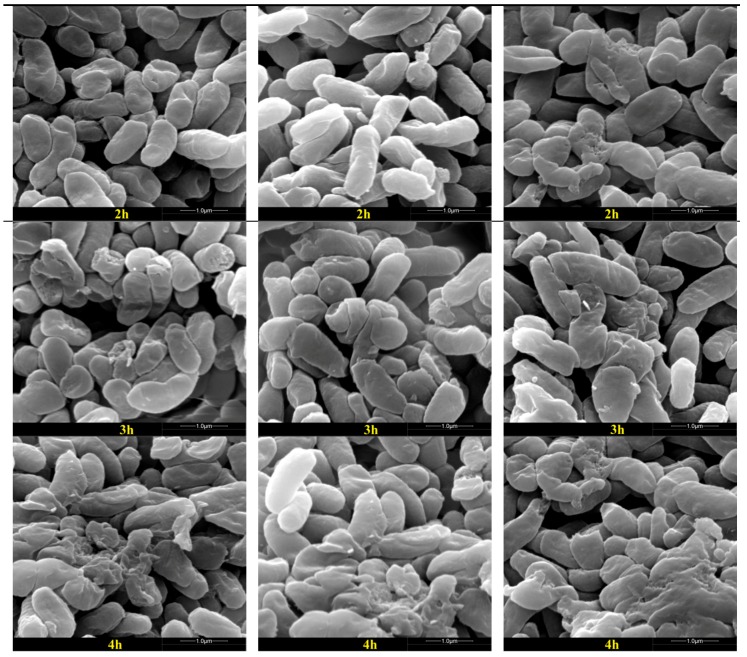
Effects of L, C, and C-L on *E. coli* CVCC 245 morphology and integrity. The first, second, and third columns were bacteria treated with 1× minimal inhibitory concentrations (MICs) L, C, and C-L, respectively. The lines from top to bottom were bacteria untreated (C group) and treated on 1, 2, 3, and 4 h.

**Figure 3:**
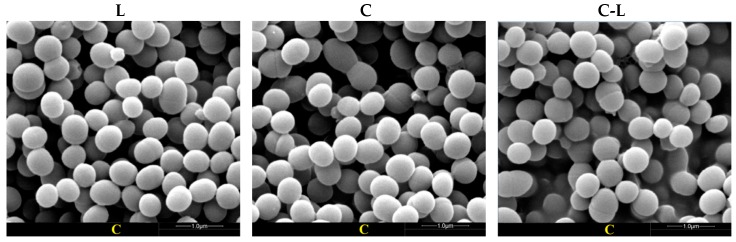

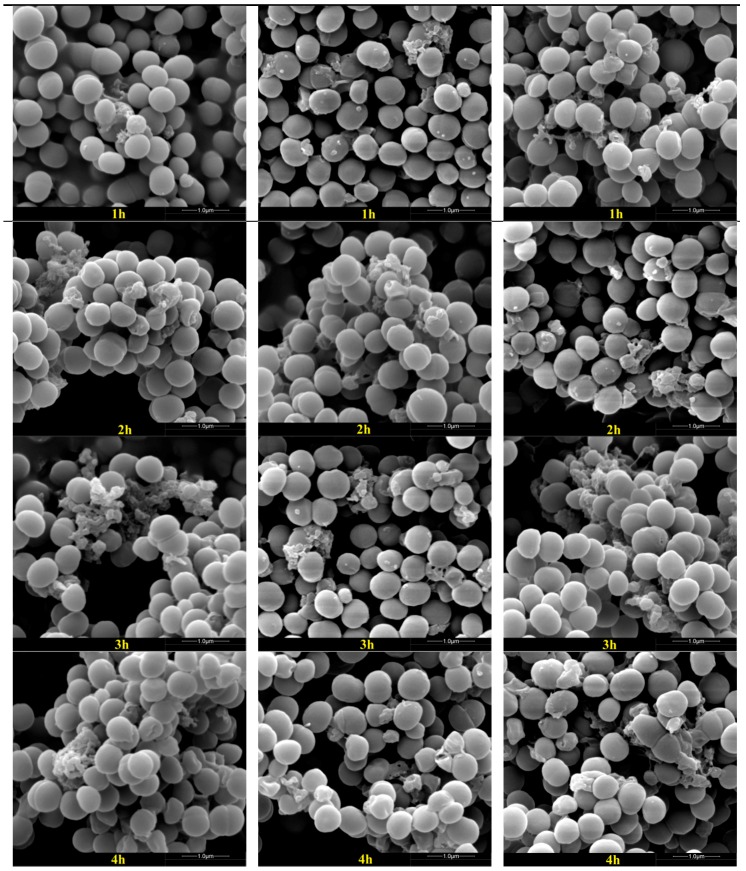
Effects of L, C, and C-L on *S. aureus* ATCC 25293 morphology and integrity. The first, second, and third columns were bacteria treated with 1 × MICs L, C, and C-L, respectively. The lines from top to bottom were bacteria untreated (C group) and treated on 1, 2, 3, and 4 h, respectively.

Scanning electron microscopy (SEM) allowed us to directly observe the external surface of the bacteria after peptide treatment. The antibacterial properties of C, L, and C-L were studied by observing the integrity of the treated bacterial cell surface structure. As shown in Figure 2 and Figure 3, some debris was observed in the surface of *E. coli* CVCC 245 and *S. aureus* ATCC 25923 after treatment with AMPs (C, L, and C-L) at MICs for 1 h, and more cellular debris and surface structure damage were observed after treatment for 2, 3, and 4 h, however, the untreated *E. coli* and *S. aureus* showed bright and smooth surfaces (Figure 2 and Figure 3). Furthermore, varying degrees of collapse or disfiguration were observed in bacterial cells treated with peptides for 2 h or more.

### 2.5. Synergistic Assay

To test the efficacy of synergism, C-L was used to treat pathogens in combination with several antibiotics. As shown in Table 5, fractional inhibitory concentration (FIC) indexes of the combination groups “C-L + thiamphenicol” and “C-L + penicillin G” against *E. coli* CVCC 245 were higher than those of the “C-L + chloramphenicol”, “C-L + neomycin sulfate”, and “C-L + kanamycin” groups (*p* < 0.01), while the FIC index of the “C-L + penicillin G” group against *S. aureus* ATCC 25923 was the highest among all the groups (*p* < 0.01). FIC indexes ≤0.5 were observed in the combination of C-L with chloramphenicol (0.375), thiamphenicol (0.188), and neomycin sulfate (0.250) against *S. aureus* ATCC 25923 and with neomycin sulfate (0.313) against *E. coli* CVCC 245; 0.5 < FIC index ≤ 1 was observed in the combination of C-L with kanamycin (0.750) against *S. aureus* ATCC 25923 and chloramphenicol (1.00) against *E. coli* CVCC 245, respectively. Neither a synergistic nor antagonistic effect was observed in other combinations (FIC values ranged from 1 to 2).

## 3. Discussion

AMPs have been considered to be potential antimicrobial alternatives to traditional antibiotics. The antimicrobial mechanism of AMPs is totally different from traditional antibiotics. Antibiotics interfered with the inner biosynthesis of proteins, RNA, DNA, peptidoglycan, and folic acid [1], however, AMPs were reported to be less prone to drug resistance because their mechanisms were largely related to the interaction with bacterial cell membranes [34,35].

Over the decades, many efforts have been made to elevate the potency of AMPs against pathogenic agents and eliminate their undesirable cytotoxicity to eukaryotic cells. Hybridizing different AMPs is one of the successful approaches to obtain novel AMPs with increased antimicrobial activity but minimized cytotoxicity [3,10,16,17,18]. As reported previously, C had been frequently used as the parental peptide in many hybrid peptide designs due to its high α-helical conformation and broad antimicrobial activity [10,12,16]. However, almost no study about L in hybrid peptides was reported, even though it is a well characterized AMP with potential antibacterial, antiviral, and anticancer activities [23,24]. In the present study, the hybrid peptide C-L was designed by combining the N-terminal amphipathic α-helix fragment of C and the core antimicrobial fragment of L. Antibacterial and TKC data demonstrated that the hybrid peptide C-L was more active against both gram-positive and gram-negative bacteria than parental peptides (C and L). Additionally, C-L did not exhibit cytotoxicity, which was in agreement with studies of other hybrid peptides, such as Me (1–13)-LL37 (17–30) [9], Mdc-Hly [3], HP (2–9)-M (1–12) [36], CA (1–8)-ME (1–12) [17,18], CA (1–8)-MA (1–12) [10,17], and CA (1–7)-ME (2–9) [14].

Generally, the antimicrobial activity of AMPs depended on their structural and physicochemical properties. In the present study, C-L had a higher net charge, α-helical content, and hydrophobicity than parental AMPs (C and L). It was believed that almost all cationic peptides kill bacteria by initial electrostatic interacting with anionic membrane, followed by penetrating into the lipid bilayer, forming channels or pores, and consequently leading to cell lysis and death [37,38]. C and L had been observed to exert their antibacterial activity by forming pores in lipid bilayers with transmembrane helices via anion selective channel and carpet-type mechanisms [19,26,39,40,41]. During the process of sterilization, the higher positive charges were reported to correlate with stronger activity by augmenting the bonding force between the AMPs and the bacterial cell membranes [29,30]. The α-helices structure allows AMPs to potently interact with negatively charged membrane [31,32]. Therefore, in present research, the increased antibacterial activity of the hybrid peptide C-L may be attributed to changes in structural and physicochemical properties (higher net charge and α-helical content). Moreover, as a resulting benefit from the no hemolysis feature provided by the first eight residues of C [12,13,14,15], C-L was able to selectively disrupt bacterial surface structure while causing little injury to sheep blood cells.

In the present study, some cellular debris was observed on the surface of the bacterial cells treated with AMPs (C, L and C-L), indicating that the peptides could cause disruption of bacterial cells surface structure. Though in our study *S. aureus* ATCC 25923 and *E. coli* CVCC 245 were almost completely killed by C, L, and C-L within 5 h of incubation, C-L was found to be more effective than C or L in the first 3 h. The three peptides mentioned above provide protection against a wide variety of pathogens and show great potential to regulate intestine microflora as a promising candidate for antibiotics. However, excessive proteolysis may limit the therapeutic application of exogenous L in the gut. Incubation of L with 1 ng/mL trypsin at 37 °C for 3 h resulted in approximately 50% activity loss of the initial amount of L, and with 10 and 100 ng/mL resulted in almost 100% inactivation [42]. According to the preliminary assessment in our ongoing study (unpublished), similar sensitivity of C and C-L to trypsin was found when compared with L. Given this situation, C-L would have a better effect in the process of regulating intestine microflora than C and L because of its higher efficiency in a shorter amount of time.

Various AMPs and antibiotics were tested for synergistic effects against different bacterial strains [11,43] (e.g., the combination of nisin with daptomycin, ciprofloxacin or vancomycin resulted in increased antibacterial activity against both methicillin-resistant and methicillin-susceptible *S. aureus* strains [44]; the combination of CA (1–7)-ME (2–9) with β-lactam or ciprofloxacin resulted in increased antibacterial activity against *Acinetobacter baumannii* ATCC 19606 and *Acinetobacter baumannii* 04–01 [14,45]). In the present study, the FIC values calculated from the checkerboard experiments revealed that C-L acted synergistically with chloramphenicol, thiamphenicol, or neomycin sulfate against *S. aureus* ATCC 25923, and with neomycin sulfate against *E. coli* CVCC 245. The results were in agreement with previous synergy studies between AMPs and antibiotics [11,14]. Although the mechanism of this positive interaction remains unclear, we concluded the potential reasons were as follows: on the one hand, antibiotics promoted AMPs to stride over the outer membrane and then act on an intracellular basis, on the other hand, AMPs could penetrate the cell membrane and allow the antibiotic to act more effectively.

## 4. Materials and Methods

### 4.1. Bacterial Strains and Antibiotics

Six pathogenic bacterial strains were purchased from China Veterinary Culture Collection (CVCC, Beijing, China). The strains were *E. coli* K88, *E. coli* CVCC 245, *S. aureus* CVCC 26003, *S. aureus* ATCC 25923, *Micrococcus luteus* (*M. luteus*) CVCC 28001, and *Listeria monocytogenes* (*L. mono.*) CVCC 1599. Neomycin sulfate, chloramphenicol, thiamphenicol, kanamycin, and penicillin G were all purchased from Sigma (San Francisco, CA, USA).

### 4.2. Design, Biological Information Analysis, and Synthesis of Hybrid Peptides

Hybrid peptides derived from selected parental peptides were designed using APD2 [46]. Protein primary structure characteristics (including net charge, helical residue, and hydrophobic residue) were analyzed and predicted by Jpred 4 [47] and SSpro [33] online. The selected hybrid peptide and parental peptides were synthesized by 9-fluorenylmethoxycarbonyl solid-phase synthesis chemistry and purified by a reverse-phase semi-preparative high performance liquid chromatography (SBS, Shenzhen, China), and then stored at −80 °C until analysis.

### 4.3. Determination of Minimal Inhibitory Concentrations (MICs)

The antibacterial activities of the peptides were characterized by MICs. Bacterial strains at exponential phase were diluted to the concentration of 5 × 10^5^ CFU/mL with Mueller-Hinton (MH) broth medium, and 180 µL culture was dispensed into each well of a 96-well microtiter plate. Susceptibility tests were performed by two-fold standard broth microdilutions of the test peptides (C-L, C, and L) according to the Clinical and Laboratory Standards Institute guidelines. Aliquots (20 µL) of peptide dilution were mixed with 180 µL bacterial suspensions, and the mixture was assayed with Bio-Rad 3700 plate reader by monitoring optical density at 600 nm after 16–18 h incubation at 37 °C. Each analysis was performed in triplicate. The lowest concentration (highest dilution) required to prevent the growth of bacteria was regarded as the MICs [2].

### 4.4. Hemolytic Assay

The hemolytic activities were evaluated by measuring the peptide concentrations that caused 50% hemolysis of sheep erythrocytes at 540 nm (A540 nm) [17,48]. Sheep erythrocytes with 10 mM phosphate buffered saline (PBS)/0.1% (*v*/*v*) Triton X-100 (Sigma) added were used as negative/positive control. Experiments were performed in triplicate.

### 4.5. Antibacterial Studies

Indicator strains *S. aureus* ATCC 25923 and *E. coli* CVCC 245 were cultured overnight and then diluted with MH to 10^8^ CFU/mL. The bacterial suspensions were mixed with C, L, and C-L (total volume, 200 µL; final concentrations of 1 × MICs) respectively, incubated at 180 rpm and 37 °C. At 0, 1, 2, 3, 4, and 5 h, an aliquot (10 µL) of the culture was collected, diluted, and plated on an MH plate. Colonies were counted at 24 h and the original concentration was calculated. Each analysis was performed in triplicate.

### 4.6. SEM Analysis of Bacterial Cells

*E. coli* CVCC 245 and *S. aureus* ATCC 25923 cell suspensions (1 × 10^8^ CFU/mL) were incubated with C-L (final concentrations of 1 × MICs, respectively) at 37 °C, 180 rpm. Four milliliters of mixture were taken at 0, 2, and 4 h and centrifuged at 10,000× *g* for 10 min—the supernatant was discarded. The bacterial cells were washed with PBS three times, fixed with 2.5% glutaraldehyde at 4 °C overnight, washed twice with PBS again, and postfixed for 2 h with 1% OsO_4_. The bacterial pellets were dehydrated with a grades series of ethanol and dried cells were coated with gold and observed under a scanning electron microscope (Quanta 200, FEI, Hillsboro, OR, USA).

### 4.7. Synergy Assays

Studies on synergistic effects between antibiotics and C-L on controlling pathogens (*E. coli* CVCC 245 and *S. aureus* ATCC 25923) were performed with the checkerboard titration method. The drugs (C-L and antibiotics) were set in a series of concentrations (ranged from 1/16× MICs to 3× MICs). Experiments were performed in triplicate. The FIC index for combinations was calculated according to the equation: FIC index = FIC_A_ + FIC_B_ = A/MIC_A_ + B/MIC_B_, where A and B are the MICs of drug A and drug B in the combination, respectively, MIC_A_ and MIC_B_ are the MICs of drug A and drug B, respectively, and FIC_A_ and FIC_B_ are the FICs of drug A and drug B, respectively. The FIC index was interpreted as follows: ≤0.5, synergy; 0.5 to 1.0, addition; 1.0 to 4.0, indifference; and ≥4.0, antagonism [49].

### 4.8. Statistical Analyses

Experiment data were analyzed by one-way ANOVA with general liner model procedure of SPSS (version 20.00, SPSS Institute Inc., Chicago, IL, USA). Statistically significant effects were further analyzed and means were compared using Duncan’s multiple range test. Statistical significance was determined at *p* < 0.05.

## 5. Conclusions

In conclusion, a novel hybrid peptide C-L with antibacterial activity that could cause disruption of cell surface structure was designed and tested. The hybrid peptide C-L did not show cytotoxicity to sheep blood cells. Due to the synergistic effect of hydrophobic N-terminal fragment of cecropin C and the core antimicrobial fragment of L, peptide C-L showed excellent antibacterial properties. The results indicated that the hybrid peptide C-L could serve as a promising candidate for pharmaceutical agents.

## Figures and Tables

**Table 1:** Amino acid sequence and molecular weight (*M*_W_) of C-L, L, and C determined by Matrix-assisted laser desorption ionization (MALDI) mass spectrometry.

Peptide	Amino Acid Sequence	Theoretical *M*_W_	Measured *M*_W_
C-L	KWKLFKKIFKRIVQRIKDFLRN	2905.70	2905.60
L	LLGDFFRKSKEKIGKEFKRIVQRIKDFLRNLVPRTES	4490.57	4490.60
C	KWKLFKKIEKVGQNIRDGIIKAGPAVAVVGQATQIAK	4010.60	4010.82

**Table 2:** Structure parameters of the peptides predicted by online software Jpred 4 and SSpro.

Peptide	Structure	Net Charge	Helical Residue (%)	Hydrophobic Residue (%)
C-L	α-helical	+8	90.9	45
L	α-helical	+6	81.1	35
C	α-helical	+7	81.1	45

**Table 3:** The minimal inhibitory concentrations (MICs) and hemolytic activity (HC_50_) of C-L, C, and L against six bacterial strains and sheep erythrocyte cell.

Bacteria Strains		Antimicrobial Peptide			*p*-Value
		C	L	C-L	
MIC (µg/mL)	*E. coli* K88	64 ± 0.72 ^a^	61 ± 1.78 ^b^	8 ± 0.33 ^c^	<0.01
	*E. coli* CVCC 245	4 ± 0.28 ^c^	84 ± 1.54 ^a^	7 ± 0.29 ^b^	<0.01
	*S. aureus* CVCC 26003	128 ± 7.57 ^a^	36 ± 1.55 ^b^	7 ± 0.28 ^c^	<0.01
	*S. aureus* ATCC 25923	198 ± 10.1 ^a^	58 ± 1.68 ^b^	2 ± 0.03 ^c^	<0.01
	*L.* *mono.* CVCC 1599	64 ± 2.99 ^a^	13 ± 0.43 ^b^	2 ± 0.06 ^c^	<0.01
	*M. luteus* CVCC 28001	154 ± 5.49 ^a^	90 ± 1.43 ^b^	16 ± 0.18 ^c^	<0.01
HC_50_ (µg/mL)	Sheep erythrocyte cell	169 ± 15.1 ^b^	32 ± 0.68 ^c^	221 ± 3.27 ^a^	<0.01

MICs, minimal inhibitory concentrations; *S. aureus*, *Staphylococcus aureus*; *E. coli*, *Escherichia coli*; *L. mono.*, *Listeria monocytogenes*; *M. luteus.*, *Micrococcus luteus*; ^a,b,c^ Means with different superscripts within the same row differ (*p* < 0.01).

**Table 4:** The sterilization rate of L, C, and C-L on *S. aureus* ATCC 25923 and *E.* *coli* CVCC 245.

Bacteria Strains	Time (h)	Antimicrobial Peptides			*p*-Value
		C	L	C-L	
Viable *S. aureus* concentration (×10^8^ CFU/mL)	1	0.54 ± 0.007 ^b^	0.90 ± 0.020 ^a^	0.43 ± 0.017 ^c^	<0.01
	2	0.40 ± 0.035 ^b^	0.53 ± 0.026 ^a^	0.30 ± 0.012 ^c^	<0.01
	3	0.21 ± 0.014 ^a^	0.23 ± 0.011 ^a^	0.18 ± 0.020 ^b^	0.014
	4	0.09 ± 0.005	0.09 ± 0.002	0.08 ± 0.003	0.079
	5	0.01 ± 0.0005	0.01 ± 0.0003	0.01 ± 0.0001	0.298
Viable *E. coli* concentration (×10^8^ CFU/mL)	1	0.44 ± 0.028 ^a^	0.44 ± 0.027 ^a^	0.29 ± 0.015 ^b^	<0.01
	2	0.30 ± 0.021 ^b^	0.35 ± 0.014 ^a^	0.16 ± 0.026 ^c^	<0.01
	3	0.15 ± 0.014 ^a^	0.14 ± 0.020 ^a^	0.05 ± 0.003 ^b^	<0.01
	4	0.06 ± 0.002	0.05 ± 0.002	0.05 ± 0.003	0.061
	5	0.01 ± 0.0002	0.01 ± 0.003	0.01 ± 0.0001	0.659

^a,b,c^ Means with different superscripts within the same row differ (*p* < 0.05).

**Table 5:** Synergistic interaction of C-L with five antibiotics.

Combination Group	Fractional Inhibitory Concentration (FIC) Index					*p*-Value
	C-L + Chloramphenicol	C-L + Thiamphenicol	C-L + Neomycin Sulfate	C-L + Penicillin G	C-L + Kanamycin	
*E. coli* CVCC 245	1.00 ± 0.141 ^c^	1.50 ± 0.001 ^a^	0.313 ± 0.001 ^d^	1.50 ± 0.003 ^a^	1.25 ± 0.002 ^b^	<0.01
*S. aureus* ATCC 25923	0.375 ± 0.005 ^c^	0.188 ± 0.002 ^e^	0.250 ± 0.004 ^d^	1.13 ± 0.005 ^a^	0.750 ± 0.003 ^b^	<0.01

^a,b,c,d,e^ Means with different superscripts within the same row differ (*p* < 0.05).
